# The Anti-Neuroinflammatory Effect of Fuzi and Ganjiang Extraction on LPS-Induced BV2 Microglia and Its Intervention Function on Depression-Like Behavior of Cancer-Related Fatigue Model Mice

**DOI:** 10.3389/fphar.2021.670586

**Published:** 2021-05-28

**Authors:** Songwei Yang, Yantao Yang, Cong Chen, Huiqin Wang, Qidi Ai, Meiyu Lin, Qi Zeng, Yi Zhang, Yan Gao, Xun Li, Naihong Chen

**Affiliations:** ^1^ Hunan University of Chinese Medicine and Hunan Engineering Technology Center of Standardization and Function of Chinese Herbal Decoction Pieces, Changsha, China; ^2^ State Key Laboratory of Bioactive Substances and Functions of Natural Medicines, Institute of Materia and Neuroscience Center, Chinese Academy of Medical Sciences and Peking Union Medical College, Beijing, China

**Keywords:** Fuzi, Ganjiang, CRF, neuroinflammation, BV2, NF-κB, Nrf2/HO-1

## Abstract

The Chinese herb couple Fuzi and Ganjiang (FG) has been a classic combination of traditional Chinese medicine that is commonly used clinically in China for nearly 2000 years. Traditional Chinese medicine suggests that FG can treat various ailments, including heart failure, fatigue, gastrointestinal upset, and depression. Neuroinflammation is one of the main pathogenesis of many neurodegenerative diseases in which microglia cells play a critical role in the occurrence and development of neuroinflammation. FG has been clinically proven to have an efficient therapeutic effect on depression and other neurological disorders, but its mechanism remains unknown. Cancer-related fatigue (CRF) is a serious threat to the quality of life of cancer patients and is characterized by both physical and psychological fatigue. Recent studies have found that neuroinflammation is a key inducement leading to the occurrence and development of CRF. Traditional Chinese medicine theory believes that extreme fatigue and depressive symptoms of CRF are related to *Yang* deficiency, and the application of Yang tonic drugs such as Fuzi and Ganjiang can relieve CRF symptoms, but the underlying mechanisms remain unknown. In order to define whether FG can inhibit CRF depression-like behavior by suppressing neuroinflammation, we conducted a series of experimental studies *in vitro* and *in vivo*. According to the UPLC-Q-TOF/MS^E^ results, we speculated that there were 49 compounds in the FG extraction, among which 30 compounds were derived from Fuzi and 19 compounds were derived from Ganjiang. Our research data showed that FG can effectively reduce the production of pro-inflammatory mediators IL-6, TNF-α, ROS, NO, and PGE_2_ and suppress the expression of iNOS and COX2, which were related to the inhibition of NF-κB/activation of Nrf2/HO-1 signaling pathways. In addition, our research results revealed that FG can improve the depression-like behavior performance of CRF model mice in the tail suspension test, open field test, elevated plus maze test, and forced swimming test, which were associated with the inhibition of the expression of inflammatory mediators iNOS and COX2 in the prefrontal cortex and hippocampus of CRF model mice. Those research results suggested that FG has a satisfactory effect on depression-like behavior of CRF, which was related to the inhibition of neuroinflammation.

## Introduction

The central nervous system (CNS) has been described as an immunologically quiescent organ that maintains the normal function of the human system, and the homeostasis of the CNS heavily relies on the balance of innate immunity (Yang and Zhou, 2019). The results of research in recent decades have suggested that deregulated CNS innate immunity made a great contribution to the onset and progression of many diseases related to the nervous system. Neuroinflammation, or the activation of the neuroimmune cells, has been identified as an etiological factor in several neurological diseases (Schain et al., 2017). Under normal circumstances, neuroinflammation is the comprehensive innate immune response of nervous tissue to restrain infection and eliminate pathogens, misfolded proteins, and cell debris, which contributes to neural tissue fix and restoration. However, in chronic neurological diseases or traumatic stress, neuroinflammation instead becomes persistent and detrimental to nerve cells (Yang and Zhou, 2019). The brain has long been accepted as an “immunologically privileged” organ, and it protects nervous tissue by blocking direct contact between peripheral materials and the nervous organ through the blood–brain barrier. Instead, the glial cells (mainly microglia and astrocytes) are the main composition of the brain and are the primary constituents of the dedicated neuroimmune system (Kanegawa et al., 2016).

The human brain is mainly composed of microglia, astrocytes, and other neuronal cells (Kanegawa et al., 2016). Microglia and astrocytes, as resident macrophages in the CNS, play pivotal roles in the innate immune response and act as the frontline defense against pro-inflammatory stimulation and exogenous toxic substances (Graeber et al., 2011; Ransohoff et al., 2015). The glia cells provide dual functionality (pro-inflammatory and anti-inflammatory) and participate in multiple physiological functions under basal or disease conditions, including steroid release, phagocytosis, cellular repair, and free radical reduction (Schain et al., 2017). However, pro-inflammatory responses stimulated by pathological factors, including reactive oxygen species and release of cytokines, may be harmful to normal neurons, causing synaptic dysfunction, neuronal death, and loss of synapses.

Microglia cells represent 10–15% of the human brain and play an important role in defending the CNS in response to exogenous toxins (Lee et al., 2018). It has been reported that microglia played a critical role in neuroprotection under normal conditions (Tremblay et al., 2011). Nevertheless, pathologically activated microglia distinctly accelerate neuroinflammatory and neurotoxic reactions through releasing many kinds of pro-inflammatory cytokines or mediators including interleukin-1β (IL-1β), interleukin-6 (IL-6), nitric oxidate synthase (iNOS), tumor necrosis factor-α (TNF-α), and cyclooxygenase-2 (COX-2) (Kirkley et al., 2017). Accumulating evidence suggests that chronic neurological diseases including Alzheimer’s disease (AD), Parkinson’s disease (PD), amyotrophic lateral sclerosis (ALS), Huntington’s disease (AD), and multiple sclerosis (MS) are closely related to neuroinflammation (Dansokho and Heneka, 2017). Multiple signaling pathways have been shown to be involved in the abnormal activation of microglia. Furthermore, Nrf2 has been validated to inhibit microglia hyperactivation by restraining the NF-κB and p38 MAPK signaling pathways (Koh et al., 2011). Further evidence showed that Nrf2 knockout caused mice to be more sensitive to the neuroinflammation stimulated by lipopolysaccharide (LPS), as demonstrated by an increase in the pro-inflammatory mediators TNF-α, IL-6, and iNOS (Innamorato et al., 2008). This means that exploring the mechanisms of the microglia inflammatory response and new intervention strategies may be effective ways to research neuroinflammation and neurological diseases.

Cancer-related fatigue has been defined as a distressing, persistent, subjective sense of physical, emotional, and/or cognitive tiredness or exhaustion related to cancer and cancer treatment that is not proportional to recent activity and interferes with usual functioning (National Comprehensive Cancer, 2003). CRF is now considered as one of the most extensive and distressing adverse effects of cancer itself and cancer treatment (Lawrence et al., 2004). The sensitivity to fatigue in a cancer patient can be elevated before the onset of cancer treatment and typically increases during chemotherapy, radiotherapy, and hormonal/biological therapies (Bower, 2014). The estimated prevalence of CRF during cancer therapies ranges from 25 to 99%, depending on the patient population, type of cancer or cancer treatment, and method of CRF assessment (Servaes et al., 2002). CRF has a detrimental impact on life, work, social relationships, and mood, causing distinct impairment in the overall quality of life during or after cancer treatment (Bower et al., 2000). Furthermore, exceeding fatigue led to a disruption in cancer treatment and predicted shorter recurrence-free survival and overall survival (Groenvold et al., 2007). Individual differences; skeletal, muscular, and mitochondrial dysfunction; peripheral immune activation and inflammation dysfunction; and neuron and central nervous system (CNS) disorders are suggested to be related to the incidence of CRF, but the etiology and mechanism of CRF are still unclear (Yang et al., 2019). In particular, the depression-like symptoms of CRF caused by the senses of extreme fatigue due to neuroinflammation have received widespread attention. Pro-inflammatory cytokines, such as IL-1β, IL-6, and TNF-α, released by tumor itself or immune cells following infection or tissue injury caused by chemotherapy, surgery, or radiation can signal to the CNS, leading to symptoms of distressing fatigue and other behavioral changes (Bower, 2014). A series of studies have indicated that neuroinflammation is closely associated with the occurrence and development of CRF. Therefore, the development of safe and effective drugs from the perspective of inhibiting neuroinflammation has become one of the primary tasks in the treatment of CRF.

An herb couple, two herbs frequently prescribed together to reduce toxicity and increase efficacy, is commonly applied in herbal formulae in traditional Chinese medicine (TCM) (Peng et al., 2013). Herb couples are much more convenient than other complex formulae without altering their essential therapeutic characteristics (Huang et al., 2011). The combination of Fuzi (Radix Aconiti Lateralis, derived from the lateral roots of *Aconitum carmichaelii* Debx.) and Ganjiang (Rhizoma Zingiberis, derived from the dry rhizome of *Zingiber officinale* Rosc.), namely, FG, is the frequently used herb couple involved in many Chinese medicinal formulae including Si-Ni-Tang and its deuterogenic formulae (Gao, 2005). Ganjiang–Fuzi decoction (GFD) has been used for over 2000 years in a 1:1 ratio of Ganjiang and Fuzi by weight, and it exhibits activities of dispelling internal cold, recovering depleted yang, and extricating patients in the cases of depletion of yang energy, which manifested as extremely cold limbs and faint pulse verging on expiry (Zhao et al., 2016). Clinical and experimental studies have shown that FG can significantly improve cardiac function, relieve pathological fatigue, and excite the nervous system, which is commonly used to treat heart failure and depression (Wang et al., 2010; Zhang and Fan, 2014). In traditional Chinese medicine, the fatigue symptoms of CRF are associated with *Yang* depletion, and the treatment of CRF should focus on *Yang* tonics, trying to improve the depression-like behavior. However, the problem of whether FG can improve the human nervous function by inhibiting the inflammatory response of the CNS and thus treat CRF has not been clarified yet.

Therefore, we conducted *in vivo* and *in vitro* experiments to explore the anti-neuroinflammatory effects of FG on LPS-induced BV2 microglia and its effects on depression-like behavior of CRF model mice to reveal the potential mechanism of FG in the treatment of CRF, aiming to provide a new study strategy for the traditional Chinese medicine FG in the treatment of CRF.

## Materials and Methods

### The Preparation of FG Extraction

*Aconitum carmichaelii* Debx (Fuzi) and *Zingiber officinale* Rosc (Ganjiang) were purchased from the Yangtianhe Pharmacy Co., Ltd. and identified by the Hunan Engineering Technology Center of Standardization and Function of Chinese Herbal Decoction Pieces (Changsha, China). A voucher specimen was deposited at the Hunan Engineering Technology Center of Standardization and Function of Chinese Herbal Decoction Pieces (Changsha, China). According to the application theory of traditional Chinese medicine, 300 g of Fuzi was immersed in a 10-fold amount of distilled water and boiled for 30 min, and 300 g of Ganjiang with 10-fold amount of distilled water was added and co-boiled for 30 min, and the extract was collected. The herb residues were boiled again with a 10-fold amount of distilled water for another 1 h, and the extract was collected again. Afterward, the collection extract was combined, filtered, and evaporated at 60°C and then freeze-dried into extract powder. The extract powder was spared and stored at −20°C.

### Chemical Component Analysis of FG Extraction

Characterization of the main chemical components in FG extraction was detected by UPLC-Q-TOF-MS^E^ (Waters Xevo G2-XS). Chromatographic separation was achieved using a Waters ACQUITIY UPLC BEH C18 column (2.1 mm × 100 mm, 1.7 μm) (Waters), with column temperature maintained at 35°C. The mobile phases consisted of 0.1% formic acid water (A) and acetonitrile (B) using a gradient elution. The flow rate was 0.4 ml/min, and the injection volume was 10 μl. The electrospray ionization (ESI) source was used to collect data in both positive and negative ion modes. The full scan setting parameters are as follows: the mass spectrum data format is continuum, the temperature of the desolvent is 600°C, the atomizing gas flow rate is 800 L/h, the capillary voltage is 2.5 kV, the ion source temperature is 120°C, the cone hole voltage is 40 V, the air curtain gas flow rate is 50 L/h, and the scanning range is 100–1200 m/z. During low energy scanning, the collision energy transfer and TRAP voltages were 4 and 6 eV, respectively. In high energy scanning, the transfer and TRAP voltages were 10 and 60–75 eV, respectively. Leucine-enkephalin (Lock Spray TM) was used as an internal standard to calibrate the mass axis in real time with a flow rate of 5 L/min. A self-building database containing mass spectrometry information of reported compounds from each herb was used for compound matching.

### Cell Culture and Drug Treatment

BV2 cells were purchased from the Cell Bank of Typical Culture Preservation Committee of Chinese Academy of Sciences (Shanghai, China) and were routinely cultured in D-MEM/F-12 medium supplemented with 10% fetal bovine serum (FBS, Gibco, United States), 100 U/ml penicillin, 100 μg/ml streptomycin (GE Healthcare Life Science, United States), and 25 μg/ml Plasmocin™ treatment (InvivoGen, United States) and then incubated in an incubator containing 5% CO_2_ at 37°C. The cells used in our experiments were up to passage 10. Cells were treated with FG extraction (200, 400, and 800 μg/ml) in the presence or absence of LPS (200 ng/ml, Sigma, United States). In some experiments, cells were pretreated with 8 μM BAY-11–7082 (Selleck, United States) for 2 h or 20 μM tin protoporphyrin (SnPP, Selleck, United States) for 1 h or transfected with Nrf2 siRNA/control siRNA for 24 h, followed by the treatment with FG extraction (400 μg/ml) and LPS (200 ng/ml).

4T1 murine carcinoma cells were obtained from the Cell Bank of Typical Culture Preservation Committee of Chinese Academy of Sciences (Shanghai, China). The 4T1 cells were routinely cultured in RPMI 1640 medium supplemented with 10% FBS (Gibco, United States), 100 U/ml penicillin, and 100 ug/ml streptomycin (GE Healthcare Life Science, United States) and incubated as monolayer cultures in humidified incubators containing 5% CO_2_ and 95% air at 37°C.

### Animals

Adult female BALB/c (10 weeks) mice weighing 20–22 g were obtained from Hunan SJA Laboratory Animal Co., Ltd. Female mice were selected for the study because of their stable food intake and body mass changes after being inoculated with cancer cells compared to male mice and because male mice often bite at the tumor site (Cosper and Leinwand, 2011; Yang et al., 2014). Mice were housed five per cage and maintained in temperature-controlled environments in a 12 h light cycle (lights on from 7:00 am to 7:00 pm) with *ad libitum* access to water and food. All experiments were performed in accordance with the current law and the Guiding Principles for the Care and Use of Laboratory Animals approved by the Hunan University of Chinese Medicine Animal Care and Use Committee.

### Cell Viability Assay

Cell viability assay was performed using a 3-[4,5-Dimethylthiazol-2-yl]-2,5-diphenyltetrazolium bromide (MTT) assay. BV2 cells were treated with FG extraction (100–1000 μg/ml) for 24 h and incubated with 200 μl MTT solution (5 mg/ml) for 4 h. Then, 150 μl dimethyl sulfoxide (DMSO) was added to dissolve dark blue formazan crystals generated by viable cells. Absorbance was evaluated using a microplate reader (Thermo Fisher Science, United States) at 540 nm after fully mixing the preparation by shaking the plate for 10 s. The absorbance of the control group was considered as 100% cell viability.

### Detection of Nitric Oxide

The level of NO in the cell culture supernatant was evaluated indirectly by measuring the content of nitrous acid (NO_2_^−^) via the Griess reaction. BV2 cells were treated with FG extraction (200, 400, and 800 μg/ml) in the presence of LPS (200 ng/ml) for 24 h. In some experiments, BV2 cells were pretreated with BAY-11–7082 (8 μM) for 2 h or SnPP (20 µM) for 1 h or transfected with Nrf2 siRNA/control siRNA for 24 h, followed by the treatment of FG extraction (400 μg/ml) and LPS (200 ng/ml) for 24 h. Then, the cell culture supernatant was collected and 50 μl culture supernatant was mixed with 50 μl Griess reagent Ⅰ and Ⅱ (Beyotime, China) and incubated at room temperature for 10 min. Absorbance was measured using a microplate reader at 540 nm. The content of NO was calculated according to the sodium nitrate standard curve.

### Evaluation of Cellular Reactive Oxygen Species

Intracellular production of ROS was evaluated using the fluorescent probe 2′,7′-dichlorofluorescein diacetate (DCF-DA). BV2 cells were treated with FG extraction (200, 400, and 800 μg/ml) in the presence of LPS (200 ng/ml) for 24 h and then stained with DCF-DA (10 μM, Solarbio Science, China) for 20 min in the incubator after being washed with phosphate-buffer saline (PBS, Gibco, United States). The cells were then further rinsed three times with PBS to remove extracellular DCFH-DA. The DCF fluorescence was detected at 488 nm (excitation wavelength) and 525 nm (emission wavelength) using a Cellometer^®^K2 fluorescent cell analyzer (Nexcelom, United States).

### Enzyme-Linked Immunosorbent Assay

BV2 cells were treated with FG extraction (200, 400, and 800 μg/ml) in the presence of LPS (200 ng/ml) for 24 h, and the cell culture supernatants were collected. The contents of the pro-inflammatory mediators were evaluated using mouse-PGE_2_ (Cusabio, China), IL-6, and TNF-α (QuantityCyto^®^ ELISA, China) ELISA kits according to the manufacturer’s instructions. Absorbance was measured using a microplate reader at 450 nm.

### Immunofluorescence

Immunofluorescence was conducted to observe the localization and expression of iNOS and COX2 in LPS-induced BV2 microglia. BV2 cells were treated with FG extraction (400 μg/ml) in the presence of LPS (200 ng/ml) for 24 h. Then, the cells were washed three times with PBS and fixed with 4% paraformaldehyde for 15 min, followed by permeabilization with 0.25% Triton-X100 for 10 min. Then, the cells were washed three times with PBS and incubated with primary rabbit anti-iNOS (1:500, abcam) or rabbit anti-COX2 (1:800, Cell Signaling Technology) antibodies in 1% bovine serum albumin (BSA) solution overnight at 4°C. Afterward, the cells were washed 3 times with PBS and incubated with secondary Alexa Flour^@^594 AffiniPure Goat Anti-Rabbit IgG (H + L) (1:200, YEASEN, China) or Alexa Flour^@^ 488 AffiniPure Goat Anti-Rabbit IgG (H + L) (1:200, YEASEN, China) antibodies for 1 h at room temperature. The cells were washed three times and mounted in 4’,6-diamino-2-phenylindole (DAPI)-containing solution (Solarbio, China) for 10 min and further washed three times with PBS. The images were observed using a fluorescence microscope (Olympus, Japan) and analyzed with NIH ImageJ software. Samples were analyzed in a blinded manner using 6–10 individual images.

### Nrf2 siRNA Transfection

The Nrf2 gene of BV2 microglia was silenced by Nrf2 small interfering RNA (Nrf2 siRNA, Santa Cruz Biotechnology, United States). BV2 cells were cultured and incubated in an incubator at 37°C until 70–80% confluent. 2 μl Nrf2 siRNA or control siRNA duplex was diluted into 100 μl siRNA Transfection Medium (Santa Cruz Biotechnology, United States). Simultaneously, 6 μl of Transfection Reagent (Santa Cruz Biotechnology, United States) was added into 100 μl siRNA Transfection Medium. Then, these two dilutions were softly mixed and incubated for 30 min at room temperature. After being washed two times with siRNA Transfection Medium, the cells were incubated with siRNA mixture for 6 h in the incubator. Afterward, the siRNA mixture was replaced with complete medium for another 18 h, followed by the treatment of FG extraction (400 μg/ml) in the presence of LPS (200 ng/ml) for an additional 24 h.

### Protein Isolation and Western Blot

We used a Cytoplasmic and Minute™ Nuclear Extraction Kit (Cat No:SC-003, Invent, United States) and a Minute™ Total Protein Extraction Kit for Animal Cultured Cells and Tissues (Cat No: SD-001/SN-002, Invent, United States) containing protease and phosphatase inhibitor tables (Roche, Basel, Switzerland) for protein extractions according to the manufacturer’s recommendations. The protein concentrations were evaluated using a BCA Kit (MultiSciences Biotech, China). Denatured protein samples were separated by 10% sodium dodecyl sulfate polyacrylamide gel electrophoresis (SDS-PAGE) and then transferred to polyvinylidene difluoride membranes (PVDF, Millipore, Temecula, CA, United States). After being blocked with 5% BSA or nonfat dry milk for 1 h at room temperature, the membranes were incubated with primary rabbit anti-iNOS, Nrf2, NF-κB/p65 (1:1000, abcam, United States), rabbit anti-COX2, IκBα, p-IκBα, PCNA, HO-1, and β-Actin (1:1000, Cell Signaling Technology, Waltham, MA, United States) antibodies overnight at 4°C. The membranes were washed three times with Tris-buffered saline and Tween 20 (TBST), followed by incubation with horseradish peroxidase–conjugated secondary antibodies (1:10,000, Cell Signaling Technology, Waltham, MA, United States) for 1 h at room temperature. The bands were measured using enhanced chemiluminescence (ECL) detection reagents (Amersham Biosciences, United States) according to the manufacturer’s requirements. Images were analyzed using Quantity One software.

### Mouse Model of Tumor Growth

A total of 5 ×10^5^ 4T1 cells (5 ×10^5^ cells in 0.1 ml PBS) were subcutaneously injected into the right fourth mammary fat pad of BALB/c mice as previously described (Baradaran et al., 2017). In the present study, all mice were euthanized 21 days after drug treatment. Body mass was monitored three times per week, and the tumor volume was calculated two times a week using calipers and according to the formula [(L×W2)/2] where L and W represent length and width, respectively. Behavior tests were performed two times per week. Except as noted below, mice were euthanized using pentobarbital sodium and blood was obtained by cardiac puncture. The gastrocnemius muscles and heart were extracted, weighted, and snap-frozen in liquid nitrogen for biochemical analysis; tumor mass was dissected and weighted; the brain was quickly dissected; and hippocampus and cortex brain tissues were snap-frozen in liquid nitrogen for further analysis.

### Oral Drug Administration

After tumor inoculation for 7 days, all of the mice were randomly divided into six groups: the control group (Cont); the tumor model group (Model); the minocycline group (Mino); and the FG low-, medium-, and high-dose groups (FG^L^; FG^M^; and FG^H^). Mice in the Mino group were provided with bottles of water supplemented with minocycline (1 mg/ml) at a dose of 50 mg/kg/day (Sigma, St. Louis, MO, United States). Mice in the FG^L^–FG^H^ groups were administrated with FG at the doses of 4, 8, and 16 mg/kg (i.g., once daily, 20 ml/kg, equivalent to 1, 2, and 4 times the clinical equivalent dose, respectively). Mice of the Cont and Model groups received normal saline in volumes equivalent to those used for intragastric administration of the drugs.

### Tail Suspension Test

The tail suspension test was performed under the directions described previously (Zheng et al., 2016). Mice were individually suspended by their tails using adhesive tape (from tip of tail for 2 cm). Testing was conducted for 6 min with the last 5 min scored for immobility. Mice that climbed on their tails were excluded from further testing. Mice were identified to be immobile when they exhibited no body movement and hung passively.

### Open Field Test

The open field test (OFT) was used for the evaluation of locomotor and exploratory activity and anxiety-like behavior as described previously (Li et al., 2016). The open field chamber was composed of a square arena (50 cm × 50 cm × 50 cm) made of black acrylic plate, brightly and evenly illuminated by 6 × 60 W lamps mounted 2 m above the arena. The area was divided into 4 central and 12 peripheral quadrants. Mice were individually placed into the center of the open field area and allowed to explore for 5 min. Time spent in the central and peripheral quadrants, total distance explored in 5 min, and the number of crossings between quadrants were recorded using a video camera and calculated using an analysis system. The open field chamber was cleaned using 70% ethanol between two experiments.

### Elevated Plus Maze Test

The elevated plus maze test was performed as a screening test for anxiety as previously described (Hellwig et al., 2016). The testing system consisted of a plus-shaped arena with two open or closed arms with an open roof, elevated 60 cm from the floor. Mice were placed at the four-arm intersection, and the activity of the mice was recorded using a video system. The respective times spent in the open or closed arms and the number of entries into the arms were recorded in 5 min.

### Forced Swimming Test

The forced swimming test was conducted for depression-like behavior assessment as previously described (Li et al., 2016). Mice were placed individually into a Plexiglas cylinder (30 cm in height and 25 cm in diameter) filled with 15 cm water (25 ± 1°C) and left for 6 min. The cylinder was cleaned thoroughly and filled with fresh water between each animal test. Mice were considered to be immobile when they floated in an upright position and made only slight movements to keep their head above the water. The movement of each mouse was videotaped, and the duration of immobility was recorded during the last 5 min of the 6-min testing period.

### Immunohistochemistry

The brain cryosections were rinsed with PBS and permeabilized with 0.2% Triton X-100 and 0.5% BSA solution (PBS) for 1 h at room temperature. The cryosections were subsequently incubated with primary Rabbit anti-Inos or Rabbit anti-COX2 antibodies overnight at 4°C. After being washed with 0.5% BSA (PBS) three times, the cryosections were incubated with biotin-conjugated anti-rabbit secondary antibody for 1 h at room temperature. The cryosections were rinsed with 0.5% BSA and incubated in an avidin-biotin complex solution for 1 h at room temperature. Then the cryosections were washed with PBS three times and incubated with 0.5 mg/ml 3,3′-diaminobenzidine (DAB) containing 0.003% H_2_O_2_. Afterward, the cryosections were sensed with PBS and mounted on gelatin-coated slides, and images were captured using a bright-field microscope.

### Statistical Analysis

All experiments were independently repeated three times and in triplicate unless otherwise declared. The data are expressed as means ± SEM. Statistical analysis was conducted using one-way analysis of variance (ANOVA), followed by post hoc Student–Newman–Keuls test for multiple comparisons. Statistical significance was accepted at *p* < 0.05.

## Results

### Phytochemical Characterization of FG Extraction

**FIGURE 1:**
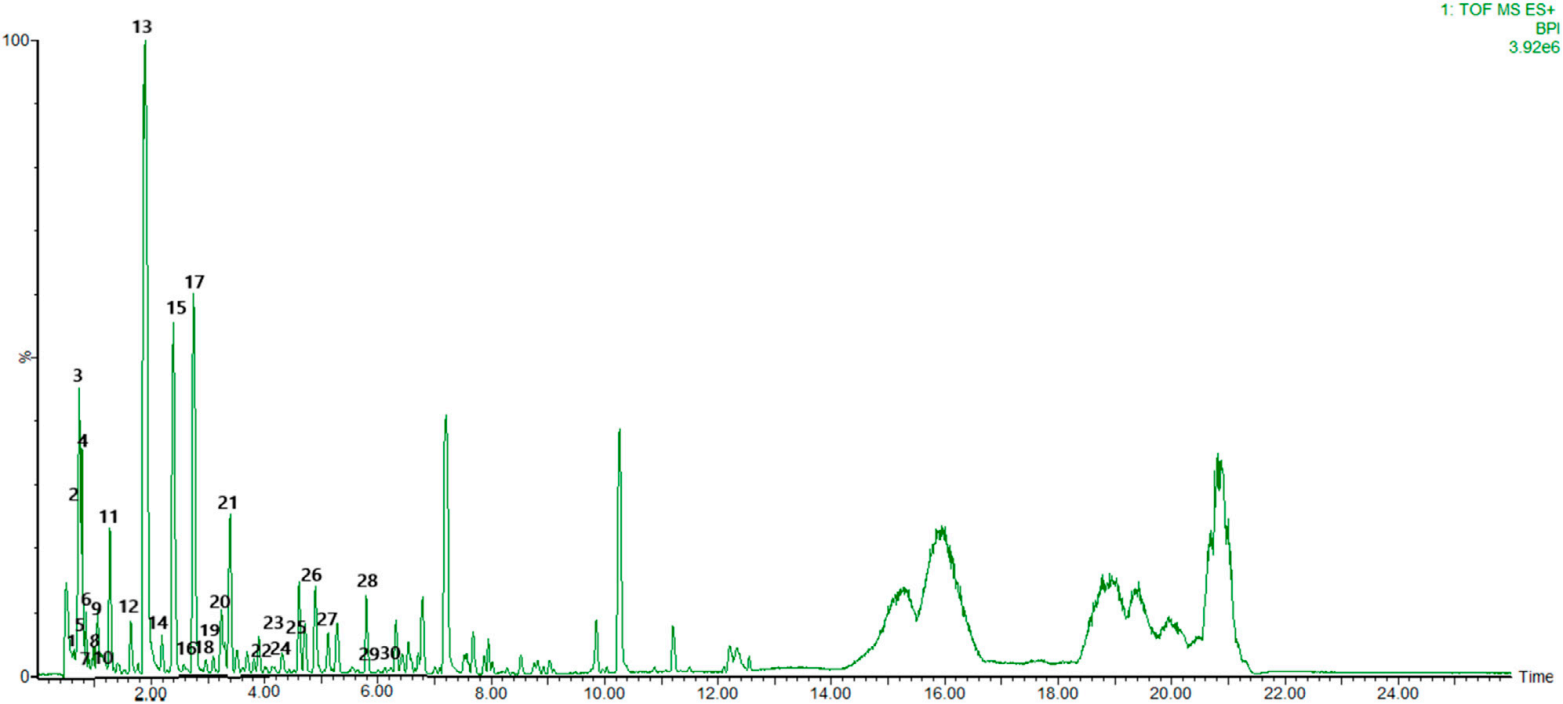
Base peak chromatogram with positive mode of the aqueous extract of FG.

**FIGURE 2:**
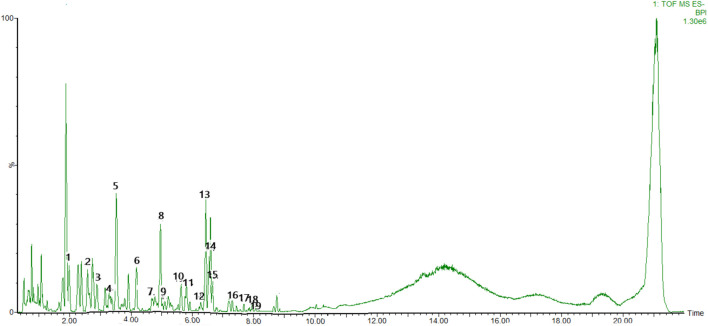
Base peak chromatogram with negative mode of the aqueous extract of FG.

To identify the main constituents of FG aqueous extraction, we analyzed the FG extraction using UPLC-Q-TOF-MS^E^. Thirty compounds were deduced from the positive spectrum, which belonged to the chemical components of Fuzi (Table 1 and Figure 1). Nineteen compounds were deduced from the negative spectrum, which belonged to the chemical components of Ganjiang (Table 2 and Figure 2). The information of 30 chromatographic peaks in the ion flow diagram in the positive ion mode was consistent with the related compounds, and all of them were derived from Fuzi. The 30 compounds included 7 amine-alcohol type C19 diterpenoid alkaloids, 13 monoester type C19 diterpenoid alkaloids, 5 diester type C19 diterpenoid alkaloids, 1 atete type C20 diterpenoid alkaloid, and 3 other types of alkaloids. In addition, 1 unknown compound was reported in the literature (Xu et al., 2015). The information of 19 chromatographic peaks in the ion flow diagram in the negative ion mode was consistent with the known compounds, and all of them belong to Ganjiang. The 19 compounds consist of 16 diphenylheptane compounds, 2 gingerols, and 1- (3, 4-dihydroxyphenyl) -5-hydroxyl-3-decanone. Furthermore, the mass spectrometry information of the remaining chromatographic peaks was inconsistent with the information of the compounds in the database and the related literature and needed further study.

**TABLE 1:** Identification of chemical constituents of Fuzi by UPLC-Q-TOF/MS^E^.

No.	T_R_ (min)	Molecular formulae	Relative molecular mass	[M + H]^+^ (Measured)	[M + H]^+^ (Theoretical)	Error (δ)	Identification
1	0.7	C_24_H_39_NO_9_	485.2625	486.2703	486.2703	1.4	Mesaconine
2	0.71	C_25_H_41_NO_9_	499.2781	500.2860	500.2859	−1.2	Aconine
3	0.73	C_24_H_39_NO_7_	453.2727	454.2805	454.2804	−2.2	Fuziline
4	0.78	C_24_H_39_NO_6_	437.2777	438.2856	438.2855	−6.4	Neoline
5	0.82	C_25_H_41_NO_8_	483.2904	484.2910	484.2910	2.5	Pseudoaconine/swatinine
6	0.87	C_24_H_39_NO_5_	421.2828	422.2906	422.2906	0.9	Talatizamine
7	0.98	C_22_H_33_NO_2_	343.2584	344.2571	344.2590	−5.5	Guanfu base H
8	0.99	C_26_H_41_NO_7_	479.2883	480.2961	480.2961	−6.2	14-O-acetyneoline
9	1.05	C_25_H_41_NO_6_	451.2934	452.3012	452.3012	1.3	Chasmanine
10	1.17	C_26_H_41_NO_6_	463.2934	464.3012	464.3012	−4.5	14-acetyltalatisamine
11	1.29	C_31_H_43_NO_11_	605.2911	606.2914	606.2914	−0.5	10-OH-benzoylmesaconine
12	1.62	C_32_H_45_NO_11_	619.2993	620.3071	620.3071	−1.0	(−) −(A-b) −14α-benzoyloxy-3α, 10β,13β,15α-tetrahydroxy-1α,6α,8β,16β,18-pentamethoxy-N-methylaconitane
13	1.94	C_31_H_43_NO_10_	589.2887	590.2965	590.2965	−0.3	Benzoylmesaconine
14	2.21	C_31_H_41_NO_7_	539.2883	540.2961	540.2961	4.4	Aconicarchamine B
15	2.38	C_32_H_45_NO_10_	603.3043	604.3122	604.3121	0.8	Benzoylaconine
16	2.57	C_33_H_45_NO_11_	631.2933	632.3017	632.3070	3.0	Mesaconitine
17	2.75	C_31_H_43_NO_9_	573.2938	574.3014	574.3016	−3.8	Benzoylhypaconine
18	2.96	C_29_H_41_NO_7_	515.2956	516.2965	516.2961	0.8	Unknown
19	3.05	C_34_H_47_NO_11_	645.3149	646.3227	646.3227	1.5	Aconitine
20	3.25	C_32_H_45_NO_8_	557.3065	558.3067	558.3067	5.4	13-deoxybenzoylhypaconine
21	3.39	C_32_H_45_NO_9_	587.3094	588.3173	588.3172	3.6	14-benzoyldeoxyaconine
22	4.01	C_31_H_43_NO_7_	541.3040	542.3118	542.3118	0.6	(−) −(A-b) −14α-benzoyloxy-N-ethyl-8β,15α-dihydroxy-1α,16β,18-trimethoxyaconitane
23	4.15	C_31_H_41_NO_8_	555.2910	556.2910	556.2910	−0.7	Dehydrated benzoylhypaconine
24	4.26	C_34_H_47_NO_12_	661.3098	662.3177	662.3176	0.6	Aconifine
25	4.6	C_33_H_45_NO_8_	583.3145	584.3223	584.3223	−1.9	(−) −(A-b) −14α-cinnamoyloxy-N-ethyl-1α,8β,15α-trihydroxy-6α,16β,18-trimethoxyaconitanebeiwutinine
26	4.92	C_32_H_45_NO_8_	571.3242	572.3223	572.3223	−3.7	14-O-anisoylneoline
27	5.12	C_33_H_45_NO_10_	615.3043	616.3122	616.3121	0.6	Hypaconitine
28	5.79	C_39_H_41_NO_11_	699.2752	700.2756	700.2758	−0.3	Trifoliolasine E
29	5.89	C_34_H_47_NO_9_	613.3251	614.3329	614.3329	0.5	(−) −(A-b) −8β-acetoxy-14α-benzoyloxy-N-ethyl-15α-hydroxy-1α,6α,16β,18-tetramethoxyaconitane
30	6.11	C_34_H_47_NO_10_	629.3200	630.3278	630.3278	3.0	Deoxyaconitine

**TABLE 2:** Identification of chemical constituents of Ganjiang by UPLC-Q-TOF/MS^E^

No.	T_R_ (min)	Molecular formulae	Relative molecular mass	[M + H]^+^ (Measured)	[M + H]^+^ (Theoretical)	Error (δ)	Identification
1	1.98	C_21_H_26_O_7_	390.1679	389.1598	389.1600	−0.5	5-hydroxy-1-(4-hydroxy-3-methoxyphenyl)-7-(3,4-dihydroxy-5-methoxy-phenyl) heptan-3-one
2	2.58	C_22_H_30_O_7_	406.1992	405.1887	405.1913	−6.4	(3 R,5S)-3,5-dihydroxy-1-(4-hydroxy-3,5-dimethoxyphenyl)-7-(4-hydroxy-3-methoxyphenyl) hepta-ne
3	2.90	C_21_H_28_O_6_	376.1886	375.1821	375.1808	3.5	3,5-dihydroxy-1,7-bis(4-hydroxy-3-methoxyphenyl) heptane
4	3.29	C_22_H_28_O_7_	404.1835	403.1750	403.1757	−1.7	3-acetoxy-5-hydroxy-1-(4-hydroxy-3-methoxyphenyl)-7-(3,4-dihydroxyphenyl) heptane
5	3.52	C_21_H_26_O_6_	374.1729	373.1654	373.1651	0.8	5-hydroxy-1,7-bis(4-hydroxy-3-methoxyphenyl)heptan-3-one
6	4.19	C_23_H_30_O_8_	434.1941	433.1848	433.1862	−3.2	3-acetoxy-5-hydroxy-1-(3,4-dihydroxy-5-methoxyphenyl)-7-(4-hydroxy-3-methoxyphenyl) hepta-ne
7	4.79	C_25_H_32_O_10_	492.1995	491.1925	491.1917	1.6	3,5-diacetoxy-1,7-bis(3,4-dihydroxyphenyl-5-methoxyphenyl)heptane
8	4.95	C_23_H_28_O_8_	432.1784	431.1708	431.1706	0.5	3,5-diacetoxy-1,7-bis(3,4-dihydroxyphenyl)heptane
9	5.11	C_24_H_30_O_9_	462.1890	461.1832	461.1812	4.3	3,5-diacetoxy-1-(3,4-dihydroxyphenyl)-7-(3,4-dihydroxy-5-methoxyphenyl) heptane
10	5.63	C_16_H_24_O_4_	280.1675	279.1596	279.1596	0.0	1-(3,4-dihydroxyphenyl)-5-hydroxy-decan-3-one
11	5.82	C_23_H_28_O_8_	432.1784	431.1708	431.1706	0.5	3-acetoxy-1,5-epoxy-3-hydroxy-1-(3,4-dihydroxy-5-methoxyphenyl)-7-(4-hydroxy-3-methoxyphenyl)heptane
12	6.28	C_23_H_28_O_7_	416.1835	415.1732	415.1757	−6.0	3,5-diacetoxy-1-(3,4-dihydroxyphenyl)-7-(4-hydroxyphenyl) heptane
13	6.43	C_25_H_32_O_9_	476.2046	475.1964	475.1968	−0.8	3,5-diacetoxy-1-(4-hydroxy -3-methoxyphenyl)-7-(3,4-dihydroxy-5-methoxyphenyl) heptane
14	6.54	C_21_H_24_O_5_	356.1624	355.1560	355.1545	4.2	1,7-bis-(4-hydroxy-3-methoxyphenyl)-4-hepten-3-one
15	6.66	C_24_H_30_O_8_	446.1941	445.1861	445.1862	−0.2	3,5-diacetoxy-1-(3,4-dihydroxyphenyl)-7-(4-hydroxy-3-methoxyphenyl) heptane
16	7.32	C_17_H_24_O_4_	292.1675	291.1573	291.1596	−7.9	[5]-gingerdione
17	7.69	C_26_H_34_O_9_	490.2203	489.2108	489.2125	−3.5	3,5-diacetoxy-1-(4-hydroxy-3,5-dimethoxyphenyl)-7-(4-hydroxy-3-methoxyphenyl) heptane
18	7.96	C_25_H_32_O_8_	460.2073	459.2016	459.2019	−0.7	1,5-epoxy-3-hydroxy-1-(3,4-dihydroxy-5-methoxyphenyl)-7-(4-hydroxy-3- methoxyphenyl)heptane
19	8.07	C_19_H_30_O_4_	322.2144	321.2070	321.2066	1.2	8-gingerol

### FG Did Not Affect Cell Viability of BV2 Microglia

**FIGURE 3:**
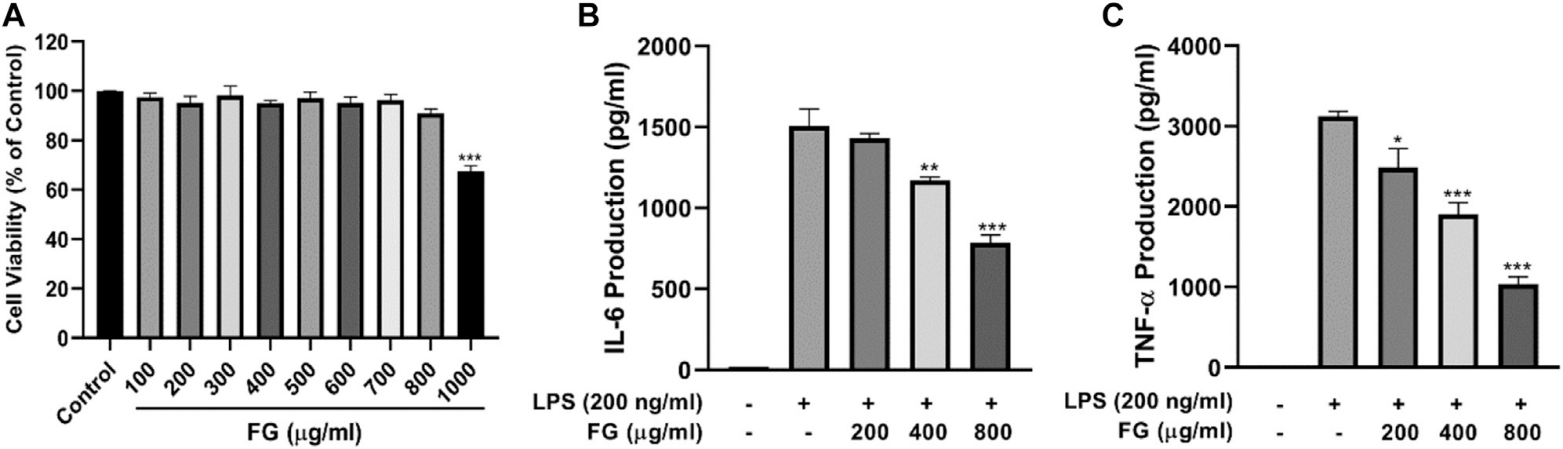
Panel **(3A):** Effect of FG on cell viability of BV2 microglia. BV2 cells were treated with 100–1000 μg/ml FG extraction for 24 h, and cell viability was evaluated using the MTT reduction assay. The results were expressed as the percentage of surviving cells in the control group. Each bar presents the mean ± SEM of three independent experiments. Panel **(3B,C)**: Effects of FG on TNF-α and IL-6 production in LPS-induced BV2 microglia. BV2 cells were treated with FG extraction (200, 400, and 800 μg/ml) in the presence of LPS (200 ng/ml) for 24 h. TNF-α and IL-6 contents in cell culture supernatants were evaluated using ELISA kits. Results are expressed as mean ± SEM of three independent experiments (^*^ *p* < 0.05, ^**^ *p* < 0.01, ^***^ *p* < 0.001).

The cell viability assay was conducted to evaluate whether the concentrations of FG used in this experiment affected the cell viability of BV2 microglia. Figure 3A shows that the treatment of FG extraction (100–800 μg/ml) for 24 h did not have a cytotoxic effect on BV2 microglia.

### FG Reduced the Secretion of TNF-α and IL-6 in LPS-Induced BV2 Microglia

To evaluate the effects of FG on the production of pro-inflammatory cytokines, the contents of TNF-α and IL-6 in the culture supernatant were measured using an ELISA kit. Figures 3B,C show an obvious increase in the levels of TNF-α and IL-6 in LPS-treated BV2 cells, and FG significantly reduced the production of TNF-α and IL-6 in a dose-dependent manner.

### FG Decreased Intracellular ROS Generation in LPS-Induced BV2 Microglia

**FIGURE 4:**
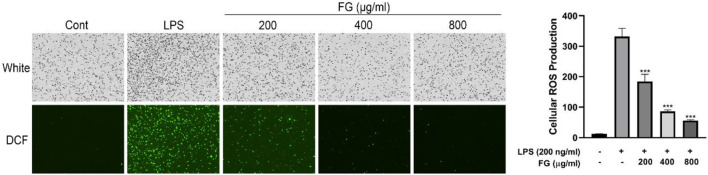
Effect of FG on cellular ROS generation in LPS-induced BV2 microglia. BV2 cells were treated with FG extraction (200, 400, and 800 μg/ml) in the presence of LPS (200 ng/ml) for 24 h. Cellular ROS level was evaluated using a DCFH-DA cellular ROS detection assay kit. Results are expressed as mean ± SEM of three independent experiments (^***^ *p* < 0.001).

To investigate the effects of FG on cellular oxidative stress in BV2 microglia, the ROS scavenging activity of FG was measured by DCFH-DA fluorescence detection. Figure 4 reveals that cellular ROS generation in BV2 cells was elevated when the cells were treated with LPS (200 ng/ml), and FG (200, 400, 800 μg/ml) obviously inhibited intracellular ROS accumulation in a dose-dependent manner.

### FG Suppressed NO Production Which Was Mediated by iNOS Expression in LPS-Induced BV2 Microglia

**FIGURE 5:**
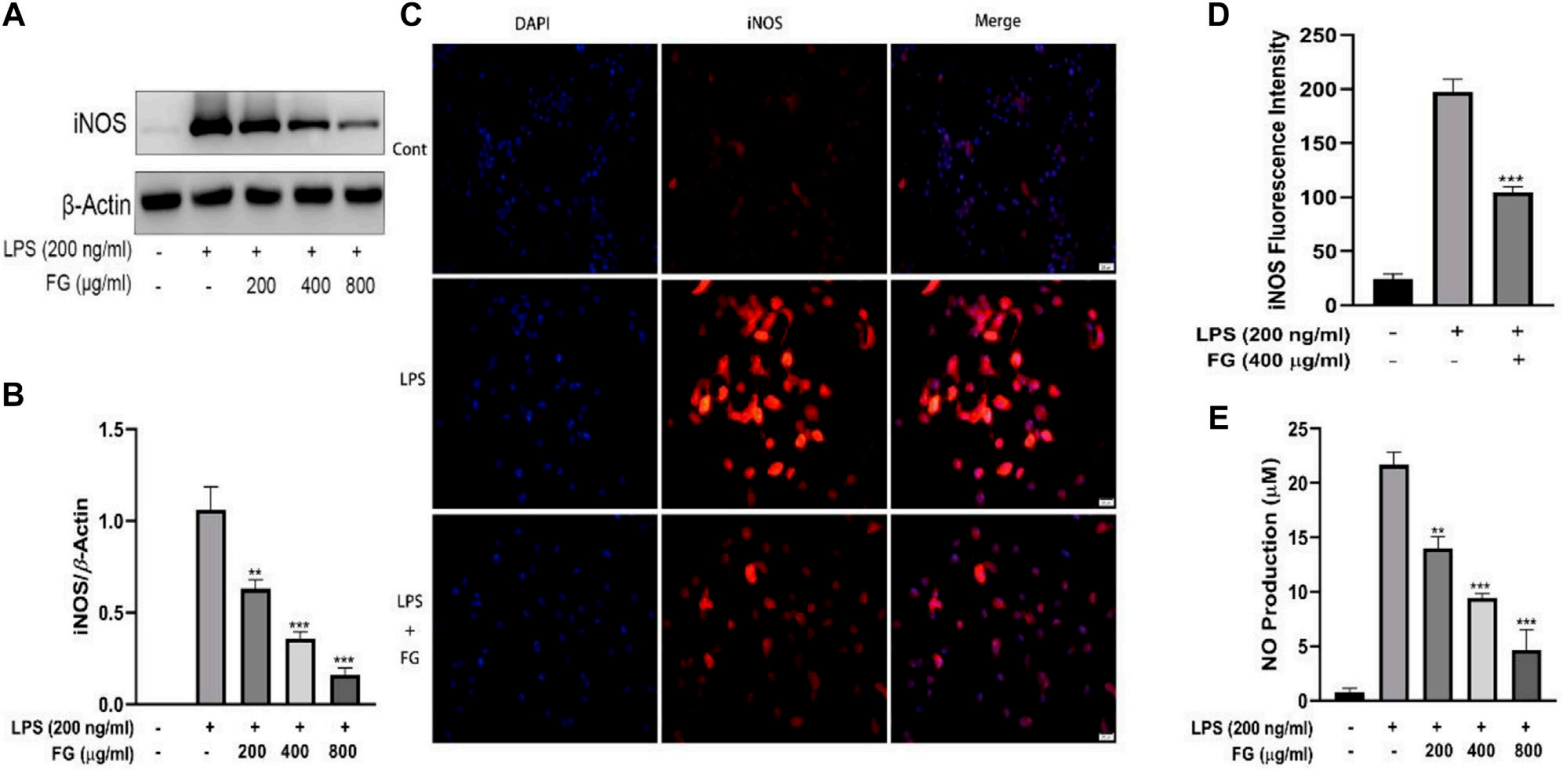
Effects of FG on iNOS and NO generation in LPS-induced BV2 microglia. BV2 cells were treated with FG extraction (200, 400, and 800 μg/ml) in the presence of LPS (200 ng/ml) for 24 h. Expression and localization of iNOS were evaluated by Western blot and immunofluorescence **(A–D)**. NO content in the cell culture supernatant was detected using a Griess assay kit **(E)**. Results are expressed as mean ± SEM of three independent experiments (^**^ *p* < 0.01, ^***^ *p* < 0.001).

To evaluate the effect of FG on iNOS-mediated NO production, which causes a toxic effect on neurocytes and results in severe neurological inflammation, the content of nitrous acid (NO_2_^−^) was measured using the Griess reaction and the expression and localization of iNOS were evaluated by western blot and immunofluorescence. Figures 5A,B show that the expression of iNOS in BV2 cells was increased when the cells were stimulated by LPS (200 ng/ml), and FG extraction (200, 400, 800 μg/ml) significantly reduced iNOS expression in a dose-dependent manner. Figures 5C,D show that the fluorescence intensity of iNOS in BV2 cells was increased by LPS (200 ng/ml) stimulation and then decreased after the treatment of FG extraction (400 μg/ml). Figure 5E shows that the content of NO in LPS-induced BV2 cells was elevated, and FG extraction (200, 400, and 800 μg/ml) suppressed NO production in a dose-dependent manner.

### FG Decreased PGE_2_ Secretion Which Was Mediated by COX2 Expression in LPS-Induced BV2 Microglia

**FIGURE 6:**
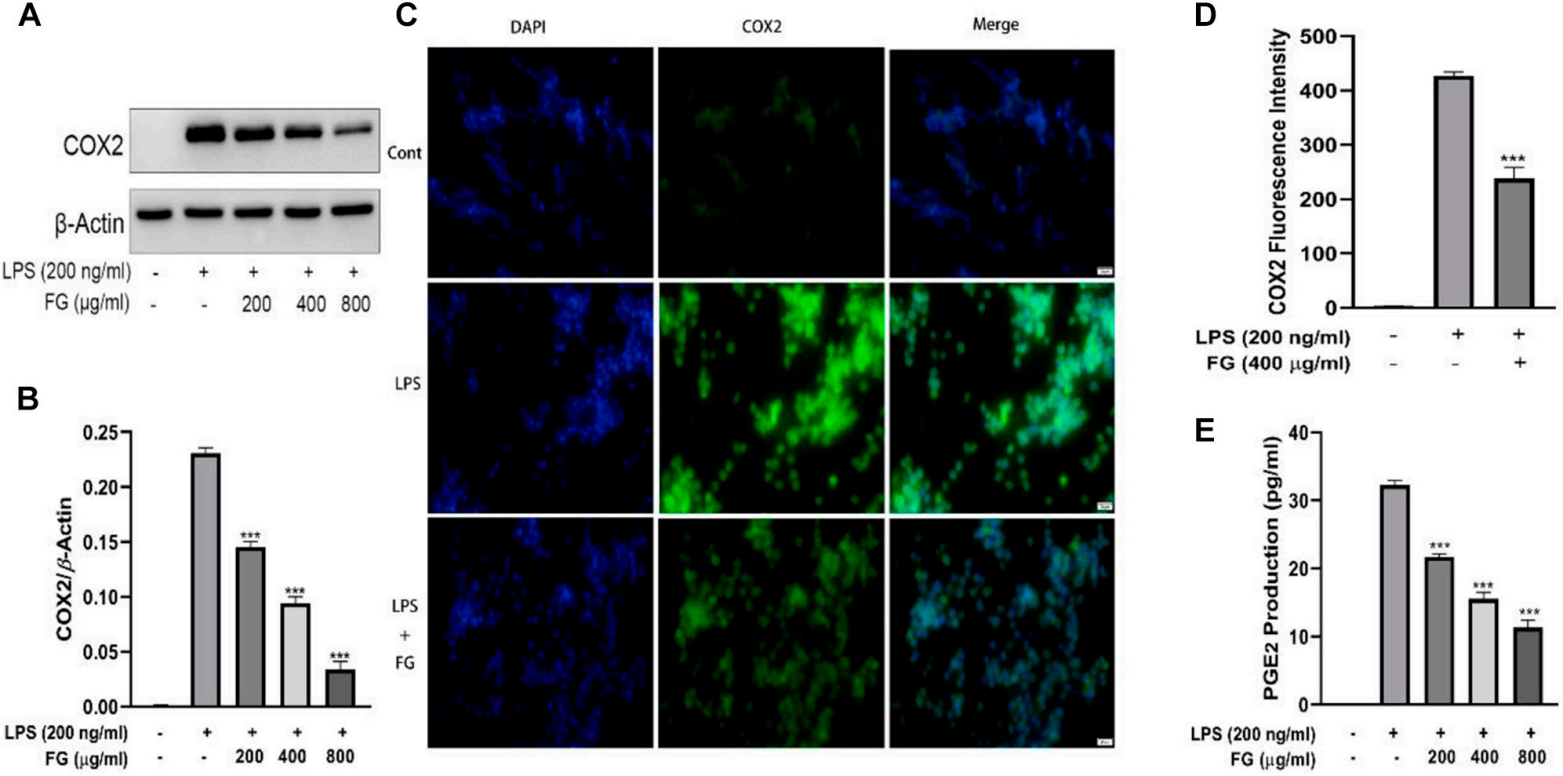
Effects of FG on COX2 and PGE_2_ production in LPS-induced BV2 microglia. BV2 cells were treated with FG extraction (200, 400, and 800 μg/ml) in the presence of LPS (200 ng/ml) for 24 h. Expression and localization of COX2 were detected by Western blot and immunofluorescence **(A–D)**. PGE_2_ level in the cell culture supernatant was detected using an ELISA kit **(E)**. Results are expressed as mean ± SEM of three independent experiments (^***^ *p* < 0.001).

To detect the effect of FG on COX2-mediated PGE_2_ secretion, which induces nerve cell damage *via* inflammatory activation, the accumulation of PGE_2_ was evaluated using an ELISA kit and the expression and localization of COX2 were measured by western blot and immunofluorescence. Figures 6A,B show that the expression of COX2 in BV2 cells was elevated under the stimulation of LPS (200 ng/ml), and FG extraction (200, 400, and 800 μg/ml) significantly decreased COX2 expression. Figures 6C,D show that LPS (200 ng/ml) enhanced the fluorescence intensity of COX2 in BV2 cells, and FG extraction (400 μg/ml) inhibited COX2 fluorescence intensity. Figure 6E shows that the production of PGE_2_ in LPS-induced BV2 cells was increased, and FG extraction (200, 400, and 800 μg/ml) suppressed the secretion of PGE_2_ in a dose-dependent manner.

### The Anti-Inflammatory Effects of FG Were Connected to the Inhibition of the NF-κB Signaling Pathway in LPS-Induced BV2 Microglia

**FIGURE 7:**
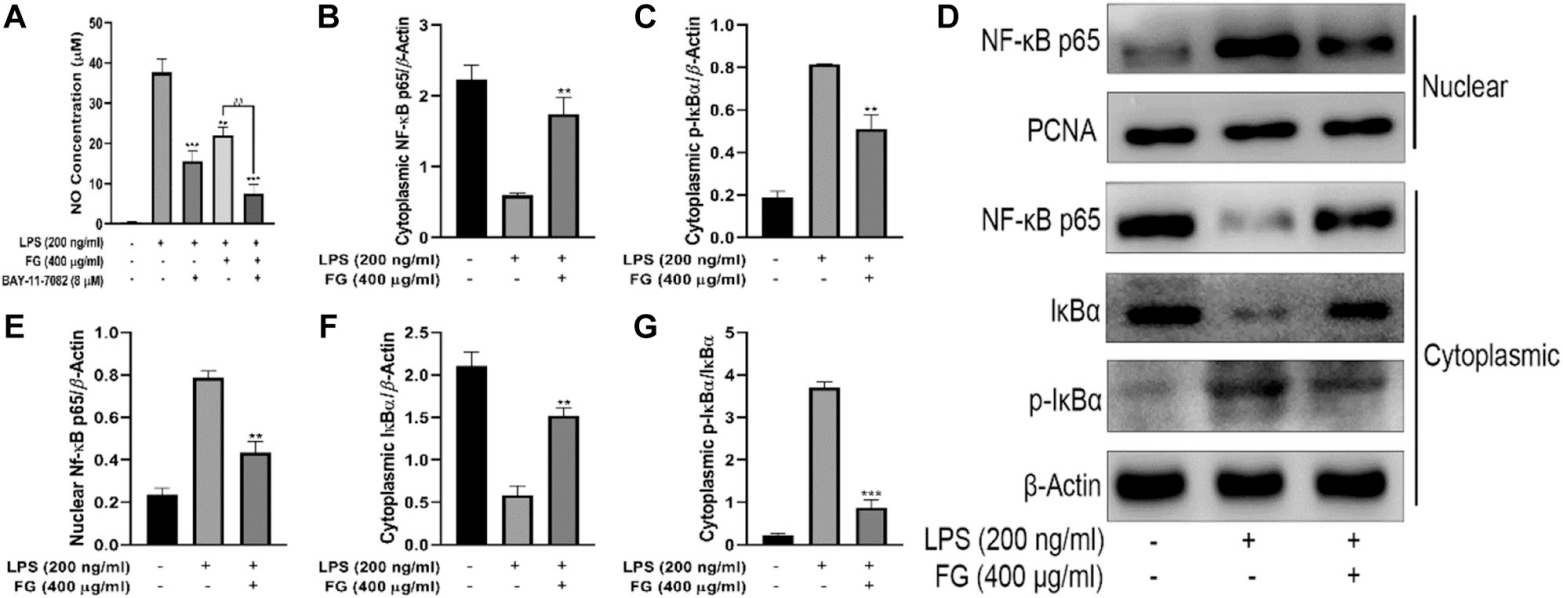
Effects of FG on the NF-κB signaling pathway in LPS-induced BV2 microglia. BV2 cells were pretreated with BAY-11–7082 (8 μM) for 2 h, followed by the treatment of FG extraction (400 μg/ml) and LPS (200 ng/ml) for 24 h. Accumulation of NO in the cell culture supernatant was measured using a Griess assay kit **(A)**. Afterward, the cells were treated with FG extraction (400 μg/ml) in the presence of LPS (200 ng/ml) for 24 h, and the nuclear and cytoplasmic protein extractions were then prepared for nuclear NF-κB/p65 and cytoplasmic IκBα and p-IκBα detection by Western blot **(B–G)**. Results are expressed as mean ± SEM of three independent experiments (^**^ *p* < 0.01, ^***^ *p* < 0.001, ^##^ *p* < 0.01).

First, we used the NF-κB inhibitor BAY-11–7082 to verify whether FG inhibits the inflammatory response of LPS-induced BV2 microglia through the NF-κB signaling pathway. Figure 7A shows that both FG extraction (400 μg/ml) and BAY-11–7082 (8 μM) reduced NO production in LPS (200 ng/ml)-treated BV2 cells, and BAY-11–7082 significantly enhanced the anti-inflammatory effects of FG, indicating that the anti-inflammatory effects of FG were closely related to the NF-κB signaling pathway. Afterward, we prepared nuclear and cytoplasmic protein extraction of LPS-induced BV2 cells to investigate the further mechanisms by which FG inhibits the NF-κB signaling pathway. Figures 7B–G show that the treatment of LPS (200 ng/ml) induced nuclear translocation of the NF-κB/p65 subunit and the phosphorylation and degradation of cytoplasmic IκBα in BV2 cells, indicating that the NF-κB signaling pathway was activated by the stimulus of inflammation. Then, we found that FG (400 μg/ml) significantly inhibited the nuclear translocation of the NF-κB/p65 subunit and the phosphorylation and degradation of cytoplasmic IκBα in LPS-induced BV2 cells, which means that the anti-inflammatory effects of FG on LPS-induced BV2 microglia were connected with the inhibition of the NF-κB signaling pathway.

### The Anti-Inflammatory Effects of FG Were Related to the Activation of the Nrf2/HO-1 Signaling Pathway in LPS-Induced BV2 Microglia

**FIGURE 8:**
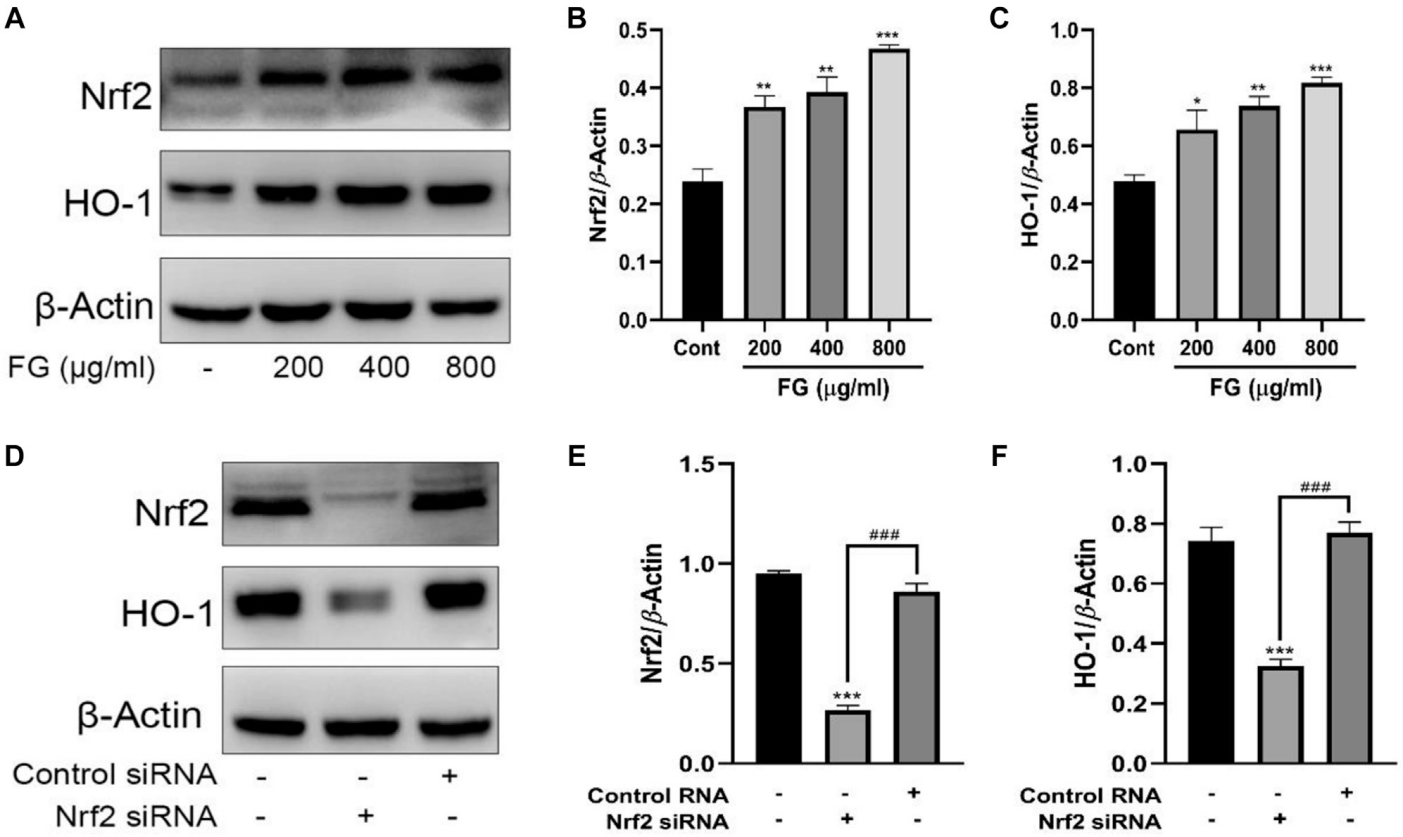
Effects of FG on the expression of Nrf2 and HO-1 in BV2 microglia. BV2 cells were treated with FG extraction (200, 400, and 800 μg/ml) for 24 h, and the expression of Nrf2 and HO-1 was measured by Western blot **(A–C)**. Then the Nrf2 gene of BV2 cells was silenced by Nrf2 siRNA or control RNA, and the expression of Nrf2 and HO-1 was evaluated by Western blot **(D–F)**. Results are expressed as mean ± SEM of three independent experiments (^*^ *p* < 0.05, ^**^ *p* < 0.01, ^***^ *p* < 0.001, ^###^ *p* < 0.001).

**FIGURE 9:**
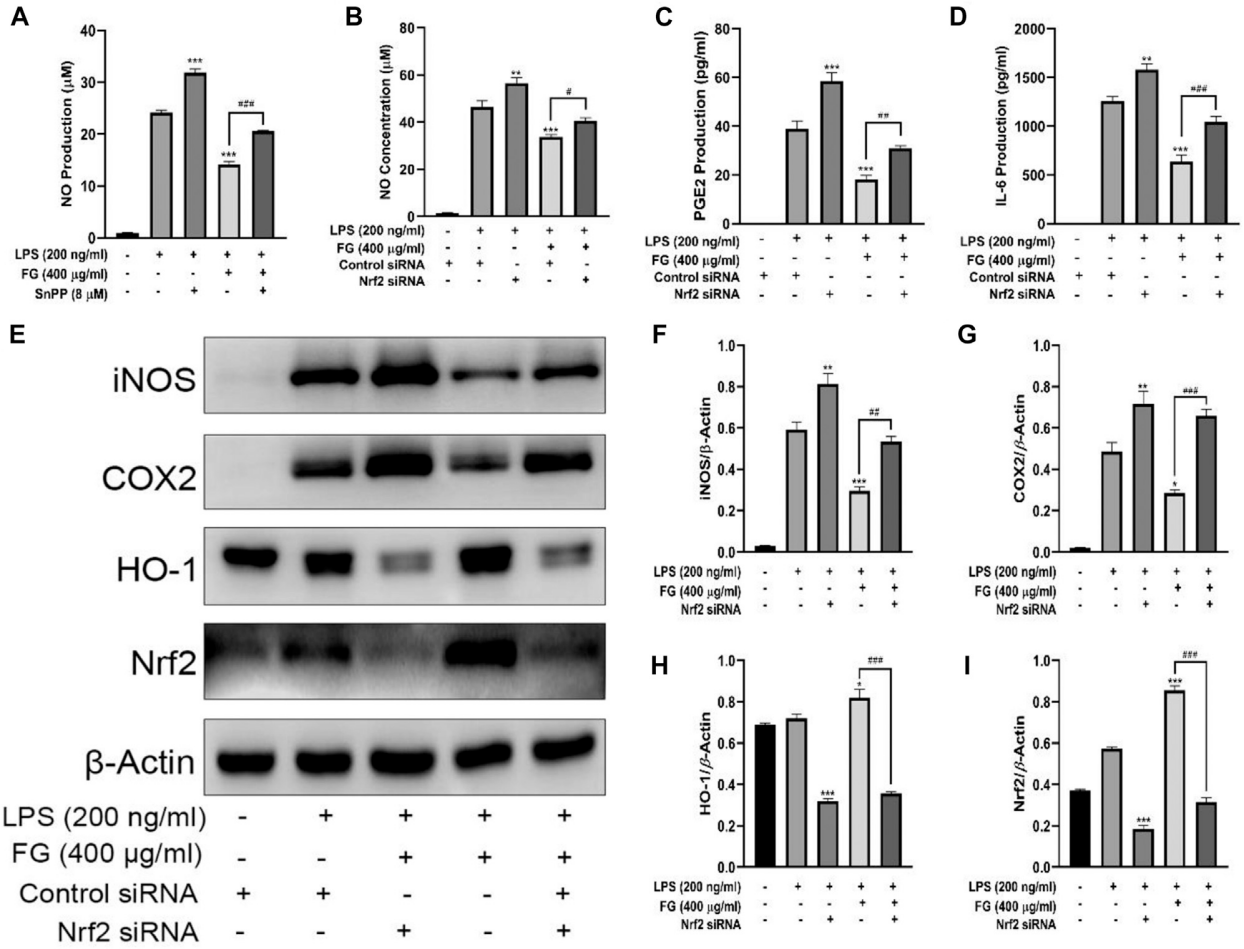
Effects of FG on the Nrf2/HO-1 signaling pathway in LPS-induced BV2 microglia. BV2 cells were pretreated with SnPP (20 µM) for 1 h, followed by the treatment with FG extraction (400 μg/ml) and LPS (200 ng/ml) for 24 h. The production of NO in the cell culture supernatant was evaluated using a Griess assay kit **(A)**. Then BV2 cells were transfected with Nrf2 siRNA or control siRNA to silence the Nrf2 gene, followed by the treatment of FG extraction (400 μg/ml) and LPS (200 ng/ml) for another 24 h. The production of NO, PGE_2_, TNF-α, and IL-6 in the cell culture supernatant was measured using Griess assay kits or ELISA kits **(B–D)**. The expression of iNOS, COX2, Nrf2, and HO-1 was detected by Western blot **(E–I)**. Results are expressed as mean ± SEM of three independent experiments (^*^ *p* < 0.05, ^**^ *p* < 0.01, ^***^ *p* < 0.001, ^#^ *p* > 0.05, ^##^ *p* < 0.01, ^###^ *p* < 0.001).

We first observed the effect of FG on the expression of Nrf2 and HO-1 in BV2 microglia and evaluated whether Nrf2 gene silencing had an inhibitory effect on HO-1 expression. Figures 8A–C show that FG (200, 400, and 800 μg/ml) could promote the expression of Nrf2 and HO-1 in normal BV2 cells, and Figures 8D–F show that the expression of Nrf2 and HO-1 was significantly decreased after Nrf2 siRNA interference silencing, indicating that FG could promote the expression of Nrf2 and HO-1 in BV2 cells, which was regulated by the Nrf2 gene. We then evaluated the relationship between the inhibitory effect of FG on the inflammatory response of LPS-induced BV2 cells and the Nrf2/HO-1 signaling pathway. Figure 9A shows that FG (400 μg/ml) reduced NO release in BV2 cells after LPS stimulation, while the HO-1 inhibitor SnPP partially reversed the inhibitory effect of FG on NO release, suggesting that the inhibitory effect of FG on NO release in LPS-induced BV2 cells was related to the regulation of HO-1 expression. Figures 9B,C show that FG (400 μg/ml) could reduce the production of NO, PGE_2_, and IL-6 in BV2 cells after LPS stimulation, indicating that the anti-inflammatory effect of FG in LPS-induced BV2 cells was related to the regulation of the Nrf2 gene. Figures 9E–I show that FG (400 μg/ml) could reduce the expression of iNOS and COX2 and increase the expression of Nrf2 and HO-1. Nevertheless, after the Nrf2 gene of the BV2 cells was silenced with Nrf2 siRNA, the inhibitory effects of FG on the expression of iNOS and COX2 and the increase in the expression of Nrf2 and HO-1 were significantly reversed, suggesting that the inhibitory effect of FG on LPS-induced BV2 cells was definitely adjusted by the Nrf2 gene. These results suggested that FG could inhibit the inflammatory response induced by LPS in BV2 microglia by activating the Nrf2/HO-1 signaling pathway.

### The Intervention Effects of FG on Depression-Like Behavior in CRF Model Mice

**FIGURE 10:**
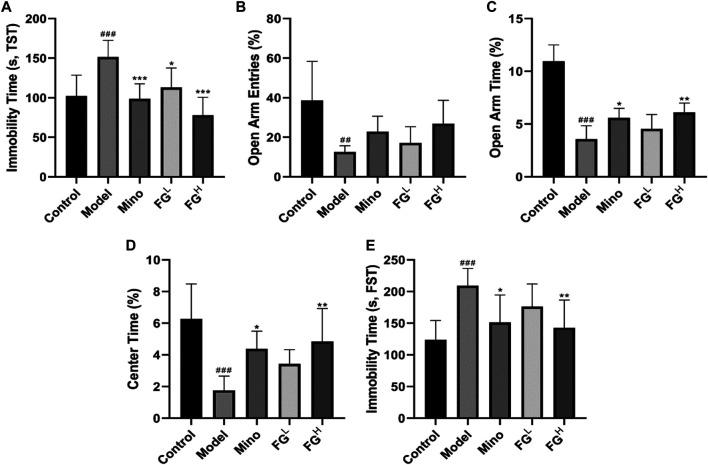
Effects of FG on fatigue-like behaviors in CRF model mice. Mice were treated with the corresponding drugs or normal saline 7 days after the inoculation of 4T1 cells. Control and Model group: the equivalent amount of normal saline; Mino group: minocycline at a dose of 50 mg/kg/d; and FG^L^ and FG^H^ group: FG extraction at the doses of 4 and 16 mg/kg/d. The fatigue-like behaviors of mice in each group were evaluated three times per week. **(A)** Locomotor activities of mice in spontaneous locomotor activity test. **(B)** Residence time of mice in rotarod fatigue test. **(C–D)** Normalized peak force measurements (PFM) and normalized average force measurements (AFM) of mice in grip strength measurements test. **(E)** Swimming time of mice in weight-loaded swimming test. Results are expressed as mean ± SD (*n* = 6–12, ^#^ *p* < 0.05, ^##^ *p* < 0.01, ^###^ *p* < 0.001 compared with control group; ^*^ *p* < 0.05 compared with model group).

To further investigate whether FG interferes with the depression-like behavior of CRF by inhibiting neuroinflammation, we inoculated BALB/c mice with the mouse breast cancer cell line (4T1) to replicate the CRF model and to evaluate the intervention effect of FG. Figure 10A shows that FG (4, 16 mg/ml) could reduce the immobility time of tail suspension in tumor-bearing mice. Figures 10B,C show that FG (16 mg/ml) could increase the residence time in the open arm of tumor-bearing mice. Figure 10D shows that FG (16 mg/ml) increased the residence time in the central region of tumor-bearing mice. Figure 10E shows that FG (16 mg/ml) decreased the immobility time of tumor-bearing mice during the forced swimming test. These results suggested that FG extraction could, to some extent, improve the depression-like behavior of CRF model mice.

### The Effects of FG on the Expression of iNOS and COX2 in the Prefrontal Cortex and Hippocampus CA1 Region of CRF Model Mice

**FIGURE 11:**
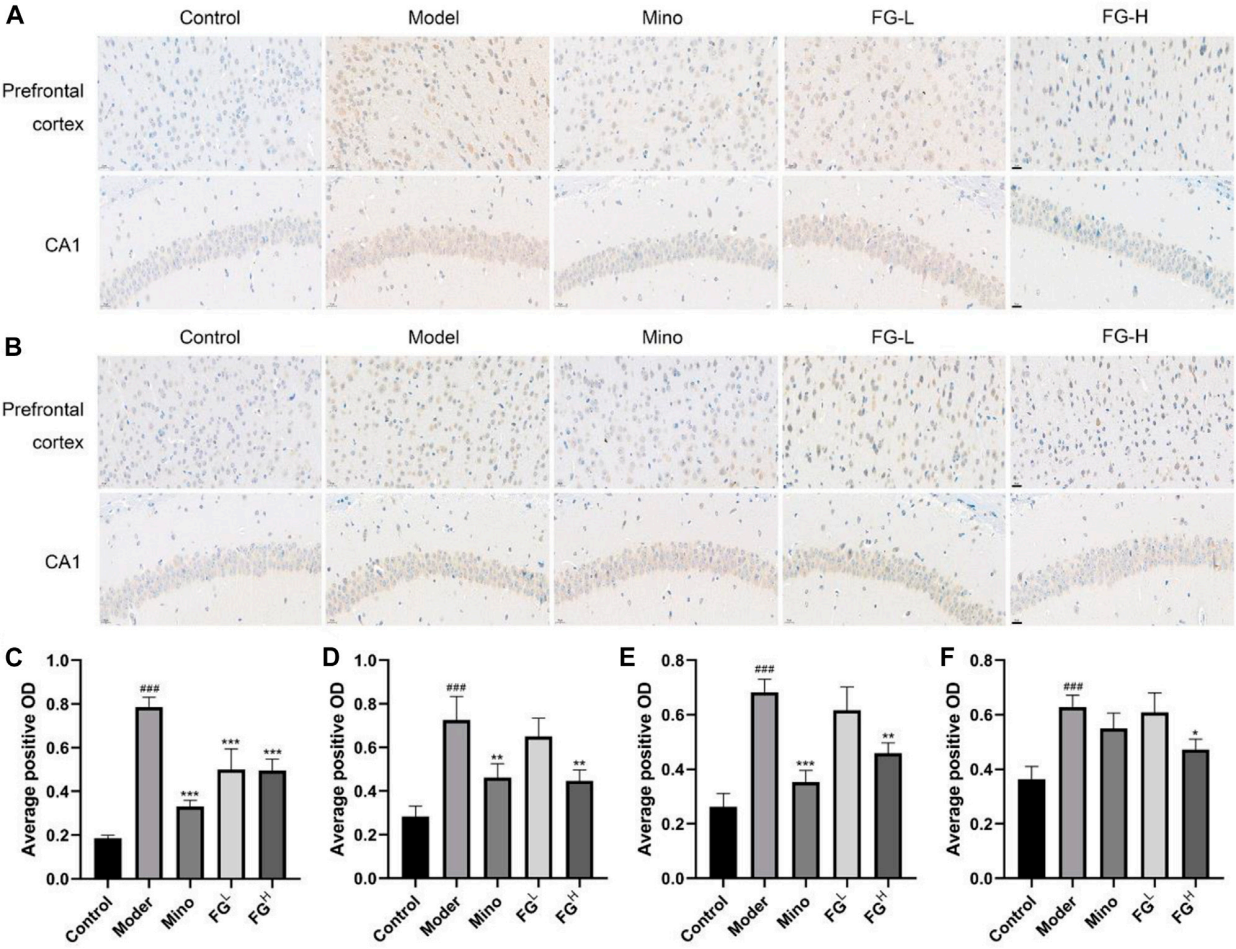
Effect of FG on the expression of iNOS and COX2 in the prefrontal cortex and hippocampal CA1 in CRF model mice (Scale bar = 20 μm). After the last administration and behavior test, the mice were deeply anesthetized with an intraperitoneal injection of sodium pentobarbital (45 mg/kg) and transcardially perfused with normal saline followed by 4% paraformaldehyde solution. The expressions of iNOS **(A, C, D)** and COX2 **(B, E, F)** in the prefrontal cortex and hippocampus CA1 region of the mice were detected by immunohistochemistry. Results are expressed as mean ± SD (^###^ *p* < 0.001 compared with control group; ^*^ *p* < 0.05, ^**^ *p* < 0.01, ^***^ *p* < 0.001 compared with model group).

Afterward, we extracted the brain tissues of CRF model mice and detected the expression of iNOS and COX2 in the prefrontal cortex and the hippocampus CA1 region of CRF model mice by immunohistochemistry. Figures 11A,C,D show that FG (4 and 16 mg/ml) could reduce the expression of iNOS in the prefrontal cortex and the hippocampus CA1 region of CRF model mice. Figures 11B,E,F show that FG (16 mg/kg) decreased the expression of COX2 in the prefrontal cortex and the hippocampal CA1 region of CRF model mice. These results indicated that FG extraction could inhibit neuroinflammation in CRF model mice, which is related to the effect of FG on depression-like behavior in CRF model mice.

## Discussion

TCMs are diffusely applied in Asian countries, especially China, Korea, and Japan (Huang et al., 2020b). Due to the precise efficacy of TCMs and the advancement of its modernization, Chinese medicine is being gradually accepted and applied by Western countries (Dobos and Tao, 2011). In traditional Chinese medicine, herb couples can provide the optimal efficacy of the formulae. Fuzi–Ganjiang (FG) is a classic herb couple among traditional medicine formulae for treating shock, heart failure, diarrhea, and other diseases characterized by Yang deficiency (Huang et al., 2020a). The theory of traditional Chinese medicine believes that Ganjiang can enhance the efficacy of Fuzi, namely, “Fuzi would not exert its heating efficacy without Ganjiang,” and multiple research studies have also found that Ganjiang and Fuzi used together can significantly heighten the pharmacological activity of those two used alone (Wen-Wen et al., 2013). Furthermore, the addition of Ganjiang could reduce the dissolution of Fuzi alkaloids, especially the acute toxic diester alkaloids, and the addition of Fuzi increased the content of gingerol compared with that in a decoction of Ganjiang alone (Huang et al., 2020a). Through the optimized UPLC-Q-TOF-MS^E^ technique, a total of 49 compounds in the FG extraction were discovered, including 30 compounds from Fuzi (Table 1 and Figure 1) and 19 compounds from Ganjiang (Table 2 and Figure 2). This method could help us to quickly identify the chemical components in FG and provide a reference for studying the pharmacodynamic substance basis of FG.

Neuroinflammation, or the activation of nerve cells, microglia, and astrocytes into pro-inflammatory phenotypes, has been considered as a pathological contributor in multiple neurodegenerative diseases (Schain and Kreisl, 2017). Several factors such as initiating insult, environmental factors, genetic background, age, and past experiences are involved in the activation of microglia and the complex neuroinflammatory pathways (Bradl and Hohlfeld, 2003; Carson et al., 2006). Abnormal microglia activation leads to the pathology of several neurodegenerative diseases including PD, AD, and MS along with psychiatric disorders such as stress, psychological fatigue, depression, and schizophrenia or metabolic syndromes including obesity, hypertension, and type 2 diabetes (Swaroop et al., 2016). Neuropathological and neuroradiological research studies suggested that the neuroinflammatory response may occur in advance of significant loss of neuronal populations in the pathological progression of neurological diseases (Frank-Cannon et al., 2009). Microglia get stimulated due to aging, oxidative stress, air pollution, and infection which causes the production of pro-inflammatory mediators such as NO, TNF-α, IL-1β, iNOS, and COX2. Excess production and accumulation of pro-inflammatory components in activated microglia turns into a risk factor for neurodegeneration via various inflammatory pathways including the PI3K/AKT and MAPK signaling pathways. Moreover, activated microglia generate superfluous intracellular ROS, which contributes to NF-κB signaling pathway activation and afterward trigger more serious neuroinflammation to promote neuronal injury and neuronal cell death (Shabab et al., 2016). Therefore, inhibiting excessive activation of microglia, reducing pro-inflammatory mediators and cytokines, and normalizing CNS function could be efficient therapeutic strategies to treat neurodegenerative diseases.

LPS is an endotoxin in the adventitial membrane of Gram-negative bacteria which causes systemic inflammatory reaction syndrome *via* loll-like receptor (TLR) signaling (Lyman et al., 2014). LPS combining to TLR4 on the microglia surface stimulates multiple signal transduction pathways, including MAPK, PI3K/AKT, and mTOR, which then lead to NF-κB activation. The activation of the NF-κB signaling pathway subsequently mediates the production of pro-inflammatory chemokines, cytokines, and inducible enzymes such as iNOS and COX2 which ultimately results in neuroinflammatory diseases (Maunsell, 2008; Park et al., 2011). Therefore, we studied the intervention effects and mechanisms of FG on LPS-induced inflammatory BV2 microglia *via* the perspective of pro-inflammatory mediator release and inflammatory signaling pathway activation. Our data showed that FG reduces the production of the pro-inflammatory mediators IL-6, TNF-α, and intracellular ROS (Figures 3, 4). NO is a free gaseous signaling molecule which adjusts the immune and nervous systems. iNOS is one of the three isoforms of nitric oxide synthase which is critical under pathological conditions; otherwise, there is no role for iNOS under normal physiological conditions in the brain (Peng et al., 2012). Microglia expressing iNOS are found in many neurological diseases, and multiple stimuli such as LPS, IL-1β, TNF-α, and IFN-γ can cause the expression of iNOS, which synthesizes NO to directly damage neuronal cells (Terazawa et al., 2013). Our research showed that FG could suppress the expression of iNOS in LPS-induced BV2 cells following the inhibitory effect of FG on the NO generation (Figure 5). COX2 is one form of the cyclooxygenases (COX) or prostaglandin H synthesis which has inflammatory functions (AD and Bosetti, 2011). COX2 catalyzes the reaction of deoxygenation of arachidonic acid (AA) to yield prostaglandin G2 (PGG2) and a peroxidase reaction which converts PGG2 to prostaglandin H2 (PGH2) and the transformed PGH2 into prostaglandin E2 (PGE_2_), which is a neuroinflammatory mediator (Hein and O’Banion, 2009; AD and Bosetti, 2011). COX2 is mainly expressed in neurons and is involved in synaptic function and memory formation. The overexpression of COX2 can directly damage neurons, and the concerned pathways are observed in neurodegenerative diseases (Shabab et al., 2016). We found that FG expressed an inhibitory effect on the expression of COX2 and reduced the synthesis of its downstream product PGE_2_ in LPS-induced BV2 cells (Figure 6). These results suggested that FG exerted significant inhibitory effects on LPS-induced BV2 microglia.

NF-κB is a critical signaling pathway which promotes microglia phagocytosis and cytokine release on the condition that TLRs recognize specific ligands to activate the inflammatory process (Lyman et al., 2014). The accommodation of NF-κB activity is dependent on its nuclear translocation during which an inhibitory molecule, IκBα, was involved. NF-κB dimmers are in moderate form in the cytoplasm and are activated by the unlocking of the inhibition of the IκB unit and the translocation of the liberated NF-κB/p65 dimmer to the nucleus (Shabab et al., 2016). Our research results showed that FG could inhibit the nuclear translocation of the NF-κB unit and the phosphorylation and degradation of IκBα in LPS-induced BV2 cells, indicating that FG exerted an anti-inflammatory role in nerve cells by inhibiting the activation of downstream inflammatory pathways. Several research studies on nitrosative/oxidative stress or inflammatory response in neurological diseases have suggested the critical role of the antioxidant pathway involving the Kelch-like ECH-associated protein (Keap1)/nuclear factor erythroid 2-like 2 (NFE2L2, namely Nrf2) (Sun et al., 2015). Heme oxygenase 1 (HO-1), an Nrf2-mediated production, is an enzyme that degrades heme to generate biliverdin, free iron, and CO. HO-1 is also a vital mediator in oxidative stress, immunoregulation, and resistance to bacterial infection (Silva-Gomes et al., 2013). We found that the HO-1 inhibitor SnPP could reduce the intervention effect of FG on the inflammatory response of LPS-induced BV2 cells, suggesting that FG could exert an antioxidant/anti-inflammatory effect by regulating HO-1 expression (Figure 9A). Previous research indicated that the Nrf2/HO-1 signaling pathway had contributed to the anti-neuroinflammatory and antioxidative progresses (Yang et al., 2020). Furthermore, the inhibition of the Nrf2/HO-1 signaling pathway might lead to the phosphorylation of NF-κB and IKKβ in LPS-stimulated BV2 cells, causing increased levels of pro-inflammatory cytokines such as TNF-α and IL-1β, suggesting that the Nrf2 signal was the regulatory factor of the NF-κB signaling pathway (Onasanwo et al., 2016; Ali et al., 2018). In addition, P38 MAPK was validated to be conducive to the stabilization of Nrf2 and the activation of HO-1 transcription, which means that p38 MAPK and ERK1/2 contribute to the Nrf2/HO-1 signaling pathway through different sites (Sun et al., 2015). MAPKs were reported to be implicated in the phosphorylation and translocation into nuclear fraction of Nrf2 (Zheng et al., 2009). The results of this research showed that the therapeutic effect of FG on LPS-induced inflammatory BV2 cells could be partly reversed by Nrf2 siRNA, suggesting that the anti-inflammatory/antioxidative effect of FG is related to the activation of the Nrf2/HO-1 signaling pathway (Figures 9B–I). However, the relationship between these three signaling pathways and neuronal inflammation and depression-like behavior of CRF still requires further experimental exploration.

Fatigue is one of the characteristics of dominating depression caused by chronic systemic inflammation and the excessive activation of neuroglia cells (Berk et al., 2013). Actually, depression and fatigue are compactly correlated in patients with cancer (Jacobsen et al., 2003). The association between fatigue and depression is intricate. Fatigue is seemingly a symptom of depression but may precipitate a depressed mood due to interference with work, social activities, leisure, and occupational activities (Bower, 2014). Several longitudinal studies suggest that depression and anxiety before cancer treatment predict CRF before, during, and after treatment (Stone et al., 2001; Miaskowski et al., 2008). In the context of inflammation, signals integrated by the hypothalamus from peripheral systems are transferred into the CNS, leading to neuroendocrine dysfunction, neural signaling alteration, and metabolic derangements. Recent evidence from both clinical and preclinical research studies indicates that elevations in pro-inflammatory cytokines, including IL-6, TNF-α, and NO, act as a common mechanism for the impact relationship of depression and fatigue in patients with CRF (Saligan and Kim, 2012; Wood and Weymann, 2013). Indeed, increased expression of pro-inflammatory cytokines in the CNS is involved in behavioral measures of fatigue, weakness, and depressed mood in the mouse model of CRF (Norden et al., 2015b). Furthermore, tumor growth itself also contributes to neuroinflammation, depression-like behavior, and fatigue prior to alterations in muscle function (Norden et al., 2015a). Our research results validated the tight association between neuroinflammation and CRF depression-like behavior, and FG can improve CRF depression-like behavior (Figure 10) by inhibiting the expression of iNOS and COX2 in the prefrontal cortex and hippocampus of CRF model mice (Figure 11), which is consistent with the results of the BV2 cell experiments above and proves the therapeutic effect of FG on CRF depression-like behavior.

Our results revealed the anti-neuroinflammatory/antioxidative effects of the herb couple Fuzi and Ganjiang (FG) on LPS-induced BV2 microglia *via* the inhibition of the production of the pro-inflammatory mediators IL-6, TNF-α, intracellular ROS, iNOS-NO, and COX-PGE_2_ and the suppression of the NF-κB and activation of the Nrf2/HO-1 signaling pathways. Moreover, the anti-neuroinflammatory effects of FG contributed to the improvement of depression-like behavior in CRF model mice. Those results will help us to further study the deeper mechanisms of FG in the treatment of CRF and its potential of clinical application, provide a novel direction for promoting the application of TCMs in modern multiple diseases, and develop new therapeutic drugs of CRF.

## Data Availability

The raw data supporting the conclusion of this article will be made available by the authors, without undue reservation.
